# Asymmetric Dimethylarginine as an Integrative Biomarker of Endothelial, Cardiometabolic and Hepatic Dysfunction in Stable Coronary Artery Disease

**DOI:** 10.3390/biomedicines14091927

**Published:** 2026-08-27

**Authors:** Lazzat Zhussupbekova, Munisakhon Makhkamova, Nargiza Nurillaeva, Dinara Nurkina, Farrukh Yuldashov, Doston Ubaydullayev

**Affiliations:** 1 Department of Internal Diseases, Astana Medical University, Astana 010000, Kazakhstan; zhussupbekova.l@amu.kz (L.Z.); nurkina.d@amu.kz (D.N.); 2 Department of Internal Diseases and Fundamentals of Preventive Medicine, Tashkent State Medical University, Tashkent 100109, Uzbekistan

**Keywords:** cardiovascular disease, metabolic disease, coronary artery disease, asymmetric dimethylarginine, endothelial dysfunction, cardiometabolic risk, MASLD, biomarker, risk stratification

## Abstract

**Background**: Coronary artery disease (CAD) frequently coexists with cardiometabolic and hepatic dysfunction, yet accessible biomarkers capturing this convergence remain limited. Asymmetric dimethylarginine (ADMA), an endogenous nitric oxide synthase inhibitor, has been proposed as an integrative marker of endothelial and metabolic impairment. This study aimed to evaluate the diagnostic value of ADMA as a unified biomarker of endothelial dysfunction (ED), cardiometabolic burden, and metabolic dysfunction-associated steatotic liver disease (MASLD) in patients with stable CAD. **Methods**: In this cross-sectional study, 298 patients with stable CAD (functional class I–II) were enrolled at two centers in Tashkent, Uzbekistan. Serum ADMA was measured by ELISA; endothelial function was assessed by flow-mediated dilation (FMD). Associations between ADMA, metabolic indices, MASLD, and CAD severity were analyzed. **Results**: MASLD was present in 48.3% of CAD patients and metabolic syndrome (MetS) in 62.4%. Patients with MASLD had more severe angina, reduced exercise tolerance, and greater ischemic burden than those without. ADMA correlated positively with BMI, waist circumference, TyG index, and TG/HDL-C ratio (all *p* < 0.001), with the TyG index showing the strongest predictive value for elevated ADMA (AUC 0.76). A dose–response relationship linked rising ADMA to worsening FMD; concentrations >160 ng/mL conferred markedly increased odds of overt ED (OR 114.8, 95% CI 6.8–1935.0) and were disproportionately prevalent in patients with MASLD (93.1% vs. 61.0%, *p* < 0.001). **Conclusions**: ADMA is markedly elevated in stable CAD and closely tracks endothelial, metabolic, and hepatic dysfunction. These cross-sectional associations position ADMA as a promising, hypothesis-generating integrative biomarker; prospective longitudinal studies are needed before its use for risk stratification in primary and cardiovascular care can be recommended.

## 1. Introduction

Coronary artery disease (CAD) remains a leading cause of morbidity and mortality worldwide, imposing a considerable burden on healthcare systems and primary care practice [1]. Despite advances in pharmacological and interventional management, the residual cardiovascular risk among patients with CAD remains high, particularly in the presence of coexisting metabolic disorders such as obesity, type 2 diabetes mellitus, and dyslipidemia [2]. These conditions frequently cluster clinically and contribute to adverse cardiovascular outcomes, underscoring the need for integrated risk assessment approaches that extend beyond traditional risk factors [3].

Endothelial dysfunction (ED) is increasingly recognized as a central pathophysiological mechanism linking CAD and metabolic abnormalities [4]. Impaired endothelial nitric oxide (NO) bioavailability leads to vascular inflammation, oxidative stress, and progression of atherosclerosis [5]. In primary care, early identification of ED may provide an opportunity for timely intervention and prevention of disease progression [6]. However, routine clinical assessment of endothelial function remains limited due to the lack of simple, reliable, and accessible biomarkers [7]. Comparable endothelial dysfunction has also been described in other vascular beds, such as pulmonary arterial hypertension, underscoring its role as a systemic rather than coronary-specific vascular mechanism [8].

**Figure 1:**
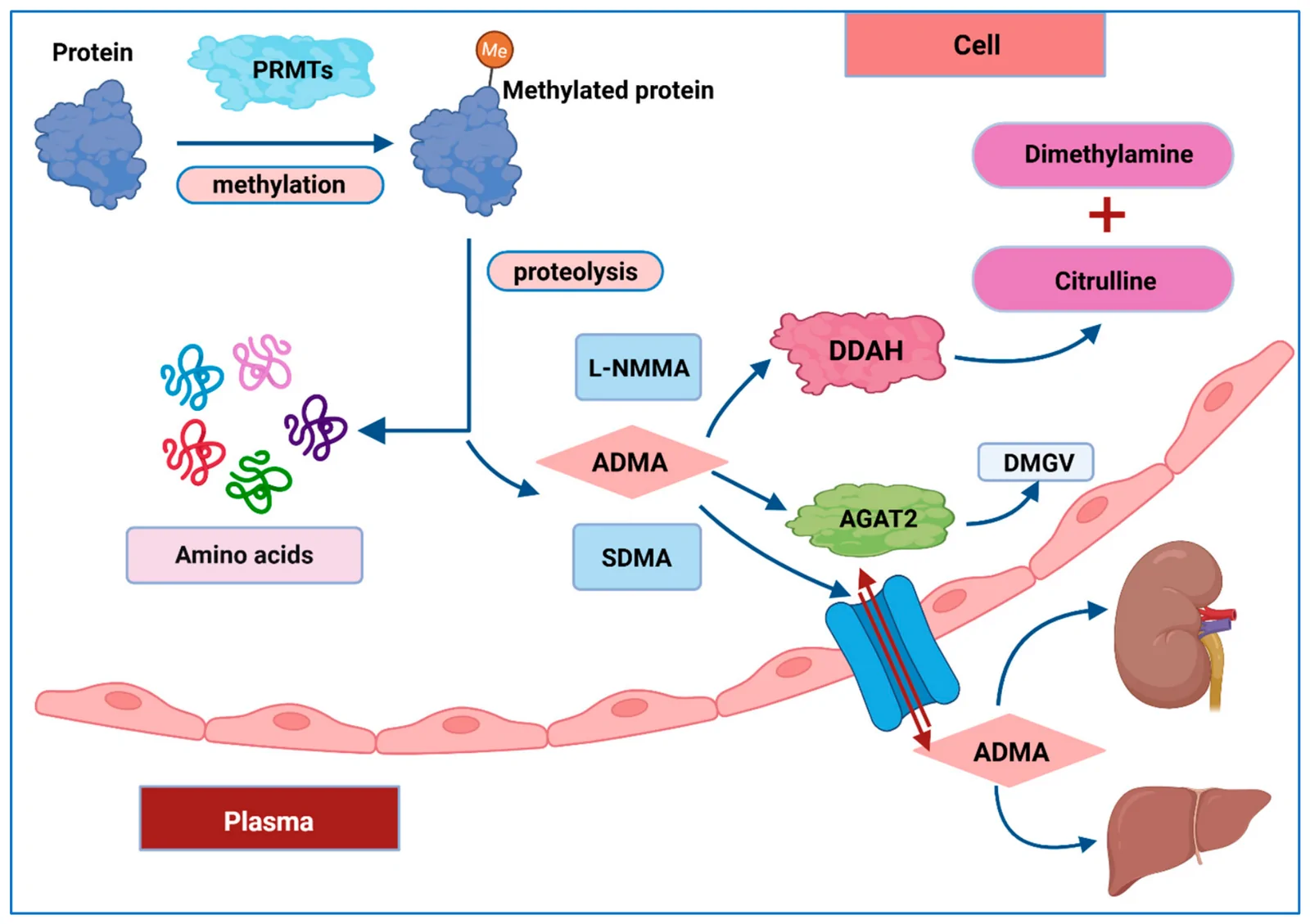
Schematic of the biosynthesis, metabolism, and excretion pathways of ADMA. (There is no copyright issue). Protein arginine methylation by PRMTs generates methylated proteins that, upon proteolysis, release free ADMA, SDMA, and L-NMMA. Intracellular ADMA and L-NMMA are largely degraded by DDAH (to citrulline and dimethylamine) or, for ADMA, partly by AGAT2 (to DMGV). Residual ADMA is exported into plasma, where it inhibits nitric oxide synthase, before being cleared by the kidney and liver. Unlike ADMA, SDMA is not a substrate for DDAH and is eliminated largely unchanged via renal excretion, whereas L-NMMA undergoes DDAH-mediated degradation in parallel with ADMA; consequently, circulating ADMA concentrations reflect the net balance of intracellular generation, DDAH- and AGAT2-mediated degradation, and renal and hepatic clearance.

Asymmetric dimethylarginine (ADMA), an endogenous competitive inhibitor of endothelial NOS, has emerged as a promising biomarker of ED [9]. Elevated ADMA levels have been associated with impaired vasodilation, increased arterial stiffness, and heightened cardiovascular risk [10]. In addition to its vascular effects, ADMA has been linked to metabolic disturbances, including insulin resistance and lipid abnormalities, suggesting its potential role as an integrative marker reflecting both endothelial and metabolic dysfunction [11]. As illustrated in Figure 1, ADMA is generated intracellularly through the post-translational methylation of arginine residues within proteins by protein arginine methyltransferases (PRMTs).

This proteolysis also releases the related methylarginines SDMA and L-NMMA; most ADMA is degraded by dimethylarginine dimethylaminohydrolase (DDAH), with a smaller fraction metabolized by alanine-glyoxylate aminotransferase 2 (AGAT2), while the unmetabolized fraction is exported into the plasma and cleared by the kidney and liver (Figure 1). Elevated plasma ADMA, reflecting an imbalance between methylarginine generation and degradation, underlies its role as a competitive inhibitor of nitric oxide synthase and a key contributor to ED [12].

Recent studies have also highlighted the interplay between cardiometabolic disorders and MASLD, further complicating risk stratification in patients with CAD [13]. This multidimensional interaction between vascular, metabolic, and hepatogenic pathways requires the identification of biomarkers capable of capturing the systemic nature of disease processes [14]. Despite growing interest in ADMA, its clinical utility as a comprehensive biomarker for simultaneous assessment of endothelial, metabolic, and subclinical atherosclerotic changes in CAD patients remains insufficiently defined [15]. In particular, there is limited evidence regarding threshold values of ADMA associated with clinically meaningful risk stratification in routine practice [16]. Therefore, the present study aimed to evaluate the diagnostic and hypothesis-generating significance of ADMA as an integrated biomarker of endothelial and metabolic dysfunction in patients with CAD, with a focus on its potential relevance to future risk-stratification research in primary care settings [17].

## 2. Materials and Methods

This cross-sectional study was conducted to evaluate the diagnostic value of ADMA as a biomarker of endothelial and cardiometabolic dysfunction in patients with CAD. The study was performed between 1 January 2024 and 1 June 2025 at two tertiary care centers in Tashkent, Uzbekistan: The Multidisciplinary Clinic of Tashkent State Medical University (TSMU) and the Department of Cardiodiabetes and Metabolic Disorders of the Republican Specialized Scientific and Practical Medical Center of Cardiology. Of the 298 patients enrolled, 104 were recruited at the Republican Specialized Scientific and Practical Medical Center of Cardiology and the remaining 194 at the Multidisciplinary Clinic of TSMU. A total of 298 patients with confirmed CAD (stable angina pectoris, functional class I–II) were consecutively enrolled. The diagnosis of CAD was established in accordance with the 2024 ESC Guidelines for the management of chronic coronary syndromes [18]. Diagnosis was based on a combination of (1) clinical evaluation, including a detailed assessment of symptoms characteristic of myocardial ischemia—exertional chest pain/discomfort and undue fatigue or breathlessness on walking—together with cardiovascular risk factor profile; (2) 12-lead electrocardiography; (3) transthoracic echocardiography; and (4) bicycle ergometry (exercise ECG stress testing), with a positive (ischemic) result defined as horizontal or downsloping ST-segment depression ≥ 1 mm (0.1 mV), measured 60–80 ms after the J point, in at least two contiguous leads, and/or reproduction of typical anginal symptoms during exercise, in accordance with standard exercise-testing criteria. The control group comprised 40 apparently healthy individuals without clinical or instrumental evidence of cardiovascular disease. This control group was recruited to characterize a healthy reference population within the same source communities and was not matched to the CAD cohort on a 1:1 basis for the present analysis; comparative statistical analyses between the CAD and control groups are not presented in this report and are addressed as a limitation below. No formal a priori sample-size or statistical power calculation was performed. The study sample comprised all consecutive eligible patients who provided informed consent during the predefined recruitment period. Accordingly, the analyses should be interpreted as exploratory, and the precision of the principal estimates is presented using 95% confidence intervals.

Metabolic dysfunction-associated steatotic liver disease (MASLD) was diagnosed in accordance with the 2023 AASLD (American Association for the Study of Liver Diseases) consensus criteria, defined as the presence of hepatic steatosis (confirmed by imaging or histology) together with at least one of the following cardiometabolic risk factors:

Body mass index ≥ 25 kg/m^2^ (≥23 kg/m^2^ in Asian populations), or waist circumference >94 cm in men and >80 cm in women.

Fasting plasma glucose ≥ 100 mg/dL (5.6 mmol/L), HbA1c ≥ 5.7%, 2 h post-load glucose ≥ 140 mg/dL, or a pre-existing diagnosis of type 2 diabetes mellitus, or treatment for type 2 diabetes.

Blood pressure ≥ 130/85 mmHg or specific antihypertensive drug treatment.

Plasma triglycerides ≥ 150 mg/dL (1.70 mmol/L) or lipid-lowering treatment.

Plasma HDL-cholesterol ≤ 40 mg/dL (1.0 mmol/L) for men and ≤50 mg/dL (1.3 mmol/L) for women, or lipid-lowering treatment.

Eligibility Criteria. Inclusion criteria: (1) confirmed diagnosis of CAD (stable angina, functional class I–II); (2) age 35–70 years; (3) provision of written informed consent. Exclusion criteria: (1) unstable angina, acute coronary syndrome, or prior myocardial infarction; (2) chronic heart failure class III–IV (New York Heart Association) or left ventricular ejection fraction < 40%; (3) severe comorbid conditions affecting endothelial function or ADMA metabolism, including chronic kidney disease (estimated glomerular filtration rate < 60 mL/min/1.73 m^2^), liver cirrhosis or advanced chronic liver disease, active malignancy, and systemic inflammatory or autoimmune disorders; (4) decompensated type 1 diabetes mellitus or poorly controlled type 2 diabetes mellitus (HbA1c > 9% at enrollment); (5) use of medications influencing ADMA metabolism within 4 weeks prior to inclusion (e.g., high-dose L-arginine or phosphodiesterase-5 inhibitors).

At baseline, all participants underwent standardized clinical evaluation, including medical history, physical examination, and assessment of CAD symptoms (frequency of angina episodes and exercise tolerance). Cardiac function was evaluated using 12-lead electrocardiography and transthoracic echocardiography. Hepatic status was assessed using a hierarchical, non-invasive algorithm: abdominal ultrasonography was performed in all patients as the first-line imaging test to confirm or exclude hepatic steatosis; the Fibrosis-4 (FIB-4) index was calculated in all patients from age, aminotransferases, and platelet count as an initial non-invasive estimate of fibrosis risk; transient elastography (FibroScan) was performed to refine fibrosis staging. The primary diagnosis of MASLD rested on ultrasonographic evidence of hepatic steatosis together with the AASLD cardiometabolic criteria described below; FIB-4 and transient elastography were used solely for non-invasive fibrosis staging in patients with confirmed steatosis, and did not themselves determine the presence or absence of MASLD.

Fasting venous blood samples were obtained from all participants. Standard biochemical analyses included liver enzymes (alanine aminotransferase and aspartate aminotransferase), lipid profile, and indices of carbohydrate metabolism. Serum ADMA concentrations were measured using enzyme-linked immunosorbent assay (ELISA) with validated commercial kits (Beijing Solarbio Science & Technology Co., Ltd., Beijing, China), in accordance with the manufacturer’s protocol. All laboratory analyses were performed under standardized conditions at the Scientific and Innovative Research Institute of Medicine, Tashkent State Medical University, ensuring analytical reproducibility and quality control. The manufacturer-stated normal reference range for this assay is [reference interval, e.g., 73–137 ng/mL, per the kit documentation]; this reference range is assay-specific and, like the diagnostic cut-offs derived in this study, has not been externally validated.

Flow-mediated dilation (FMD) of the brachial artery was assessed by high-resolution B-mode ultrasonography according to standard guidelines: baseline brachial artery diameter was recorded, followed by 5 min of forearm cuff occlusion at suprasystolic pressure and release, with diameter reassessed at peak post-occlusion dilation; FMD was calculated as the percentage change from baseline diameter. Endothelial dysfunction was defined as a priori as FMD < 7%, consistent with the FMD-based strata reported in the Section 3.

FMD and abdominal ultrasonography were performed before serum ADMA results became available. Consequently, the investigators responsible for image acquisition and interpretation were blinded to participants’ ADMA concentrations and ADMA-based analytical classification. Complete blinding to all clinical characteristics was not feasible because relevant medical information was required for the safe performance of the examinations.

Statistical analyses were performed using IBM SPSS Statistics version 23 (IBM Corp., Armonk, NY, USA); data collection and preprocessing used Microsoft Excel. Both parametric and non-parametric statistical methods were used as appropriate. Normality of continuous variables was assessed using the Shapiro–Wilk test (for subgroups of fewer than 50 patients) or the Kolmogorov–Smirnov test (for subgroups of 50 or more), together with skewness and kurtosis coefficients. Normally distributed continuous variables are presented as mean, standard deviation, and standard error, with 95% confidence intervals; between-group comparisons used Student’s *t*-test for independent samples and the paired *t*-test for related samples. Non-normally distributed variables were compared using the Mann–Whitney U-test (independent samples) or the Wilcoxon W-test (paired samples). Categorical variables were presented as frequencies and percentages and compared using the Pearson χ^2^ test; where the expected count in any cell of a contingency table was below 5, Fisher’s exact test was used instead. Correlations between continuous variables were assessed using Pearson’s r (normally distributed data) or Spearman’s ρ (non-normally distributed data), with strength of association interpreted according to the Chaddock scale. Independent predictors of ED and of ADMA elevation were identified using binary logistic regression, appropriate given the dichotomous nature of the outcomes. Variable selection used a forward stepwise procedure with the Wald statistic as the criterion for exclusion of non-significant predictors. Overall model significance was assessed using the likelihood-ratio χ^2^ test, and the proportion of variance explained was estimated using the Nagelkerke R^2^ statistic. Associations are reported as odds ratios (OR) with 95% confidence intervals (CI); some estimates derived from unadjusted category-based comparisons (rather than the multivariable models described above) show markedly wide confidence intervals reflecting small cell counts in the reference stratum. Receiver operating characteristic (ROC) curve analysis was used to evaluate the diagnostic performance of ADMA and the metabolic indices (TyG index, TG/HDL-C ratio) for identifying elevated ADMA concentrations and metabolic dysfunction. For each ROC curve, the optimal cut-off value was defined as the point providing the best combination of sensitivity and specificity (equivalent to maximizing the Youden index). The area under the curve (AUC) with standard error, 95% CI, and *p*-value is reported for each index, together with the corresponding sensitivity and specificity at the selected cut-off. A two-sided *p*-value < 0.05 was considered statistically significant.

Because no a priori sample-size calculation was performed, a post hoc power analysis was conducted for the principal between-group comparison underlying the study’s main finding—the prevalence of highly elevated ADMA concentrations (>160 ng/mL) in patients with versus without MASLD (93.1%, *n* = 134/144, vs. 61.0%, *n* = 94/154). Using a two-proportion z-test with the achieved sample sizes (n_1_ = 144, n_2_ = 154) and observed proportions, the study had >99% power (α = 0.05, two-sided) to detect this difference, indicating that the principal comparison was adequately powered. Power for secondary and more granular subgroup analyses—in particular, estimates derived from strata with small reference-category cell counts, such as the odds ratio of 114.8 for overt endothelial dysfunction at ADMA >160 ng/mL—was not separately calculated and is likely more limited, as reflected in the correspondingly wide confidence intervals reported for these estimates.

Candidate predictors entered into the stepwise selection procedure included obesity, type 2 diabetes mellitus, dyslipidemia, and accumulation of ≥3 cardiometabolic risk factors; age, sex, and smoking status were assessed for confounding but not retained given their statistical similarity between groups at baseline (Table 1). Renal function was not entered as a covariate, as patients with eGFR <60 mL/min/1.73 m^2^ were excluded a priori. Baseline medication use, including statins, antiplatelet agents, antihypertensive medications, and glucose-lowering therapy, was recorded descriptively but was not included as a covariate in the multivariable models. Therefore, the reported associations should be interpreted as unadjusted for pharmacological treatment.

The ADMA concentration categories of ≤120, 121–160, and >160 ng/mL were applied as exploratory analytical strata to characterize the graded relationship between circulating ADMA and FMD-defined endothelial dysfunction; these categories were not intended as externally validated diagnostic thresholds. Separately, the ROC-derived ADMA cut-off for identifying metabolic dysfunction was selected by maximizing the Youden index. The 120 and 160 ng/mL cut-points were adopted from thresholds proposed in a previous single-center exploratory study by members of our group [9], and have not undergone independent external validation in the present cohort or elsewhere; their use here is intended to enable comparison across studies rather than to define validated diagnostic cut-offs.

## 3. Results

### 3.1. Baseline Demographic and Clinical Characteristics

The overall study population comprised 298 patients with confirmed CAD, of whom 144 (48.3%) had concomitant MASLD (CAD + MASLD group) and 154 (51.7%) did not (CAD group). The two groups were well balanced with respect to age (58.14 ± 0.82 vs. 57.95 ± 0.80 years, *p* > 0.05) and the distribution of sex, although a numerically higher proportion of women was observed in the CAD + MASLD group (66.7% vs. 51.9%), the difference did not reach statistical significance (*p* = 0.068). No significant between-group differences were found in education level, place of residence, or employment status (all *p* > 0.05), indicating comparable socioeconomic backgrounds across groups.

Current smoking was infrequent in both groups (5.4% overall) and did not differ significantly between groups (*p* = 0.118). Notably, alcohol consumption was reported exclusively in the CAD group (24.7%) and was absent in patients with concomitant MASLD (0%), a difference that was highly statistically significant (*p* < 0.001).

With respect to hypertension severity, the proportions of patients with controlled normotension, grade I, and grade II hypertension were similar between groups (all *p* > 0.05), and no patients in either group presented with grade III hypertension. Systolic and diastolic blood pressure values, as well as left ventricular ejection fraction, were also comparable between the CAD + MASLD and CAD groups, with no statistically significant differences detected (*p* > 0.05 for all comparisons) (Table 1).

Baseline pharmacological treatment was recorded for all participants at enrollment. All patients received standard therapy for stable angina pectoris in accordance with the national clinical protocol for chronic ischemic heart disease (Ministry of Health of the Republic of Uzbekistan, Order №195 of 14 June 2024), individually dosed by the treating physician. Baseline medication use across the cohort is summarized in Table 2. Given the wide use of statins, antiplatelet agents, and antihypertensive therapy in this cohort, their potential influence on endothelial function, metabolic parameters, and ADMA concentrations is addressed as a limitation in the Discussion.

### 3.2. Clinical Manifestations and Functional Status of CAD According to MASLD Status

The majority of patients had functional class II CAD (97.3%), while only 2.7% were classified as functional class I. Disease duration was comparable between patients with and without MASLD (4.03 ± 0.15 vs. 4.10 ± 0.12 years, *p* = 0.05). Resting heart rate did not differ significantly between groups. Patients with concomitant MASLD demonstrated a more severe clinical presentation of CAD. Specifically, they reported greater angina pain intensity assessed by the visual analog scale (4.8 ± 1.2 vs. 4.1 ± 1.1 points, *p* = 0.002), a higher frequency of weekly angina episodes (2.2 ± 1.4 vs. 1.5 ± 1.2 episodes, *p* = 0.004), and a longer mean duration of angina attacks (6.8 ± 2.3 vs. 5.6 ± 2.0 min, *p* = 0.006).

Functional capacity was significantly impaired among patients with MASLD. The mean angina-free walking distance was substantially lower in the CAD + MASLD group than in the CAD-only group (310 ± 85 vs. 380 ± 95 m, *p* < 0.001). Similarly, exercise tolerance assessed by bicycle ergometry was significantly reduced in patients with MASLD (68 ± 12 vs. 98 ± 15 W, *p* < 0.001). Electrocardiographic evidence of myocardial ischemia was more frequent in the CAD + MASLD group, with ischemic ST-segment depression observed in 66.7% of patients compared with 50.6% of those without MASLD (*p* = 0.006). In addition, patients with MASLD required short-acting nitrates more frequently to control angina symptoms (2.6 ± 1.2 vs. 1.0 ± 0.7 doses per week, *p* = 0.004). Although New York Heart Association (NYHA) functional class II tended to be more prevalent among patients with MASLD, the distribution of NYHA functional classes did not differ significantly between groups (*p* = 0.11). Overall, the presence of MASLD was associated with a more symptomatic course of CAD, reduced exercise tolerance, and a higher burden of myocardial ischemia, suggesting a closer interplay between hepatic steatosis and cardiovascular dysfunction. (Table 3).

### 3.3. Cardiometabolic Characteristics

Analysis of metabolic abnormalities among patients with CAD revealed a high prevalence of metabolic syndrome (MetS) components (Table 4). Abdominal obesity was the most common metabolic abnormality, affecting 214 patients (71.8%), and represented the predominant component of metabolic dysfunction within the study population (χ^2^ = 28.356, *p* < 0.001). Reduced high-density lipoprotein cholesterol (HDL-C) levels were observed in 184 patients (61.7%), while arterial hypertension was present in 174 patients (58.4%).

Disturbances in glucose metabolism, defined as fasting plasma glucose ≥ 5.6 mmol/L or type 2 diabetes mellitus, were identified in 114 patients (38.3%). Hypertriglyceridemia (triglycerides > 1.7 mmol/L) was detected in 100 patients (33.6%). Both metabolic abnormalities were statistically significant (*p* < 0.01). Overall, MetS, defined as the presence of at least three diagnostic criteria, was diagnosed in 186 patients (62.4%) (χ^2^ = 9.188, *p* = 0.002). These findings indicate a substantial burden of metabolic dysfunction and an unfavorable cardiometabolic profile among patients with CAD.

Anthropometric assessment demonstrated significantly greater adiposity among patients with concomitant MASLD compared with those without MASLD (Table 5). Patients in the CAD + MASLD group had higher body weight (90.6 ± 2.2 vs. 80.0 ± 1.0 kg, *p* < 0.05) and body mass index (BMI) (33.6 ± 0.9 vs. 29.0 ± 0.3 kg/m^2^, *p* < 0.05). Furthermore, moderate-to-severe obesity (grades II–III) was substantially more prevalent among patients with MASLD, whereas overweight and grade I obesity predominated in patients without MASLD (χ^2^ = 38.364, *p* < 0.001).

Waist circumference and waist-to-hip ratio were comparable between groups; however, abdominal obesity was more frequent in the CAD + MASLD group than in the CAD-only group (79.2% vs. 64.9%, χ^2^ = 3.72, *p* = 0.054).

Evaluation of insulin resistance using the triglyceride–glucose (TyG) index demonstrated a high prevalence of insulin-resistant phenotypes in both groups. In the CAD + MASLD group, 54.2% of patients were classified as having a high risk of insulin resistance (TyG > 8.8), whereas 30.6% and 15.3% were categorized as intermediate- and low-risk, respectively (χ^2^ = 16.583, *p* < 0.001). A similar distribution was observed among patients without MASLD, with high insulin resistance risk identified in 55.8% of participants (χ^2^ = 28.779, *p* < 0.001). No significant between-group differences in TyG categories were detected (Pearson χ^2^ = 3.242, *p* = 0.198).

In contrast, the triglyceride-to-HDL cholesterol (TG/HDL-C) ratio revealed significant between-group differences. Although most patients in both groups were classified as having low metabolic risk, a high-risk TG/HDL-C phenotype was observed exclusively in patients with MASLD. Moreover, the distribution of TG/HDL-C categories differed significantly between groups (Pearson χ^2^ = 9.466, *p* = 0.024).

Correlation analysis demonstrated significant positive associations between serum ADMA concentrations and major markers of metabolic dysfunction. ADMA levels correlated with BMI (r_s_ = 0.42, *p* < 0.001), waist circumference (r_s_ = 0.39, *p* < 0.001), TyG index (r_s_ = 0.47, *p* < 0.001), and TG/HDL-C ratio (r_s_ = 0.44, *p* < 0.001). The strongest correlation was observed between ADMA and the TyG index.

**Figure 2:**
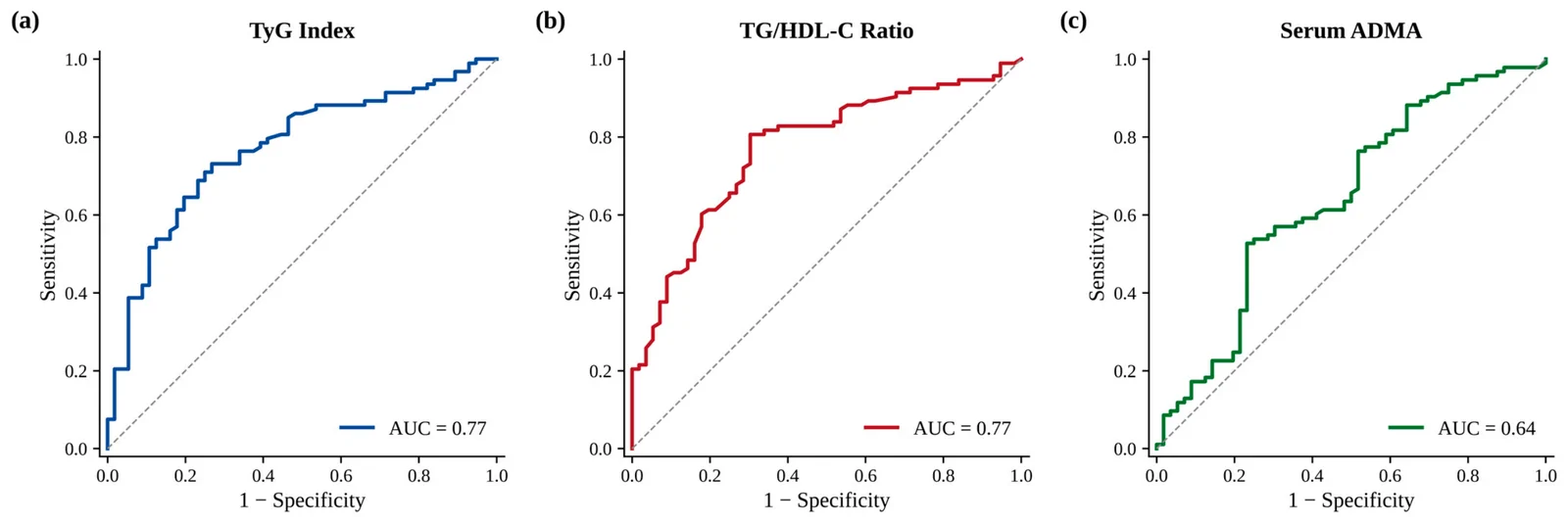
ROC curves for identification of MetS in patients with CAD (*n* = 298): (**a**) TyG index, area under the curve (AUC) = 0.77; (**b**) TG/HDL-C ratio, AUC = 0.77; (**c**) serum ADMA, AUC = 0.64. Diagonal dashed line represents the line of no discrimination (AUC = 0.50).

Receiver operating characteristic (ROC) analysis demonstrated that both metabolic indices were significant predictors of elevated ADMA concentrations. The TyG index exhibited the highest diagnostic performance, with an area under the curve (AUC) of 0.76 (95% CI 0.69–0.83, *p* < 0.001). A cut-off value of ≥8.6 yielded a sensitivity of 74.0% and specificity of 70.5% for identifying elevated ADMA levels. (Figure 2a) The TG/HDL-C ratio also demonstrated significant predictive value (AUC = 0.73, 95% CI 0.65–0.81, *p* < 0.001), with a cut-off value of ≥2.3 providing a sensitivity of 71.8% and specificity of 66.7%. These AUC values (0.64–0.77 across the indices examined here) indicate moderate, rather than excellent or strong, diagnostic discrimination and should be interpreted accordingly. (Figure 2b).

Further ROC analysis showed that serum ADMA itself possessed significant discriminatory ability for identifying metabolic dysfunction in patients with CAD. An ADMA concentration of 396.3 ng/mL represented the optimal threshold, providing a sensitivity of 52.7% and specificity of 76.8% (Figure 2c).

### 3.4. Hepatic Ultrasonographic and Fibrosis Characteristics According to MASLD Status

Abdominal ultrasonography was performed in all 298 patients to characterize hepatic morphology and confirm the presence of MASLD. Patients with CAD + MASLD showed significantly greater craniovertical dimension of the right hepatic lobe (140.33 ± 3.93 vs. 137.36 ± 2.06 mm, *p* < 0.05) and craniocaudal dimension of the left hepatic lobe (90.17 ± 0.81 vs. 83.14 ± 0.47 mm, *p* < 0.001) than the CAD-only group. Hepatic lobe thickness was likewise greater in the CAD + MASLD group, most markedly for the left lobe (63.33 ± 0.42 vs. 56.0 ± 0.23 mm, *p* < 0.001) (Table 6). Portal vein, inferior vena cava, and hepatic artery diameters were numerically larger in the CAD + MASLD group, consistent with hemodynamic remodeling accompanying hepatic steatosis.

Ultrasonographic evidence of hepatic steatotic infiltration (S1–S3) was present in 154 patients, corresponding to 51.7% of the cohort, while 144 patients (48.3%) showed no steatosis (S0). Steatosis grade was not significantly associated with CAD phenotype overall (χ^2^ = 0.168, *p* = 0.682), reflecting the clinical heterogeneity of the cohort with respect to hepatic involvement. Fibrosis was present, albeit at low grade, in a minority of patients: F0 in 32 patients (10.7%), F1 in 28 patients (9.4%), and F2 in 4 patients (1.3%). Despite the low absolute prevalence, its detection was statistically robust (χ^2^ 91.9–141.1, all *p* < 0.001).

The Fibrosis-4 index (FIB-4 index) was used as a non-invasive fibrosis-risk estimator, stratifying patients into low-probability (<1.3), intermediate-risk (1.3–2.67), and high-probability (>2.67) categories. Low fibrosis probability was observed in 102 patients (70.8%) with CAD + MASLD versus 124 patients (80.5%) with CAD only, indicating relatively preserved hepatic parenchyma in the absence of MASLD. Intermediate risk was more frequent in the CAD + MASLD group (36 patients, 25.0%) than the CAD-only group (28 patients, 18.2%), and high fibrosis probability was likewise more common in patients with MASLD (6 patients, 4.2%) than without (2 patients, 1.3%). This graded shift toward higher FIB-4 categories in the CAD + MASLD group is consistent with an early trajectory of fibrosis progression in metabolically driven hepatic injury.

Collectively, FIB-4-based staging indicates a modest but consistent gradient of fibrosis risk in patients with CAD and concomitant MASLD; the low overall prevalence of high-risk fibrosis suggests that this cohort was captured predominantly at an early stage of hepatic disease.

### 3.5. ADMA as a Marker of Endothelial Dysfunction: Association and Diagnostic Performance

To evaluate the relationship between circulating ADMA concentrations and endothelial function, patients were stratified according to predefined ADMA categories corresponding to increasing severity of ED (Table 7). Patients with ADMA concentrations ≤ 120 ng/mL were classified as having preserved endothelial function and served as the reference category. Moderate elevations in ADMA concentrations (121–160 ng/mL), corresponding to subclinical ED, were associated with an 8.6-fold higher likelihood of ED compared with the reference group (OR 8.6, 95% CI 1.8–41.2, *p* < 0.01). The strongest association was observed among patients with ADMA concentrations >160 ng/mL. This category, corresponding to overt ED, demonstrated a markedly increased odds of endothelial impairment (OR 114.8, 95% CI 6.8–1935.0, *p* < 0.001) relative to patients with normal ADMA concentrations. The extremely wide confidence interval reflects the very small number of patients with both normal ADMA and normal endothelial function in the reference.

A progressive decline in endothelial function was observed across increasing ADMA categories. While patients with normal ADMA levels generally exhibited preserved endothelial function (≥10%), those with intermediate ADMA concentrations demonstrated evidence of subclinical ED (7.0–9.9%), whereas patients with ADMA concentrations >160 ng/mL showed pronounced endothelial impairment (<6.9%). These findings describe a graded association between circulating ADMA concentrations and ED severity in this cross-sectional sample. To further characterize this association, the diagnostic characteristics of different ADMA concentration thresholds were assessed. Sensitivity, specificity, relative risk, and association coefficients were calculated to describe the discriminative performance of ADMA categories for identifying endothelial impairment. This discriminative performance increased progressively across concentration categories, with patients having ADMA concentrations > 160 ng/mL showing the strongest association with ED. The interpretation of these associations as evidence of clinical utility is addressed in the Discussion. Assessment of ADMA levels revealed a markedly more unfavorable biochemical profile among patients with concomitant MASLD compared with those without hepatic steatosis (Table 8).

In the CAD + MASLD group, none of the patients (0%) exhibited normal ADMA concentrations (≤120 ng/mL, corresponding to FMD ≥ 10%), whereas 7.79% (*n* = 12) of patients in the CAD-only group fell within this normal range. Moderately elevated ADMA levels (121–160 ng/mL, FMD 7.0–9.9%), indicative of subclinical ED, were observed in only 6.94% (*n* = 10) of patients with MASLD, compared with 31.17% (*n* = 48) of patients without MASLD. In contrast, highly elevated ADMA concentrations (>160 ng/mL, FMD < 6.9%), reflecting overt ED, predominated in the CAD + MASLD group, affecting 93.06% (*n* = 134) of patients, versus 61.04% (*n* = 94) in the comparison group. The distribution of ADMA categories differed significantly within each group (main group: χ^2^ = 53.389, *p* < 0.001; comparison group: χ^2^ = 32.909, *p* < 0.001), confirming that highly elevated ADMA was the dominant phenotype in both cohorts. However, the between-group comparison demonstrated that this skew toward severe ADMA elevation was significantly more pronounced in patients with concomitant MASLD (Pearson χ^2^ = 21.814, *p* < 0.001).

## 4. Discussion

In this cross-sectional study of patients with stable CAD, we demonstrated a high prevalence of clustered cardiometabolic risk factors, MetS, and MASLD. Importantly, these conditions were closely interrelated and collectively associated with markers of systemic metabolic burden. Within this context, ADMA emerges as a biologically plausible and clinically relevant integrative biomarker that may capture the convergence of endothelial, metabolic, and hepatogenic dysfunction.

### 4.1. Principal Findings and Clinical Interpretation

The present study confirms that patients with CAD represent a metabolically high-risk population, with more than half fulfilling criteria for MetS and nearly half exhibiting hepatic steatosis. These findings are consistent with the concept of CAD as a systemic disorder extending beyond isolated coronary pathology. The observed clustering of obesity, dyslipidemia, hypertension, and impaired glucose metabolism underscores the cumulative burden of cardiometabolic risk and its role in disease progression.

Multivariate analysis further demonstrated that obesity and the accumulation of ≥3 cardiometabolic risk factors were the strongest independent predictors of MASLD. This highlights the importance of risk factor aggregation rather than isolated abnormalities. The identified association between metabolic burden and hepatic involvement supports the concept of a shared pathophysiological axis linking adiposity, insulin resistance, and ectopic fat deposition [19]. Against this background, ADMA provides a mechanistic bridge between these domains. As an endogenous inhibitor of endothelial NOS, ADMA reduces NO bioavailability, thereby promoting ED, vascular inflammation, and atherogenesis [20]. The elevation of ADMA in patients with cardiometabolic abnormalities suggests that it reflects not only vascular impairment but also systemic metabolic dysregulation.

### 4.2. ADMA as an Integrative Biomarker

A key implication of this study is the positioning of ADMA as an integrative biomarker capable of capturing the interplay between cardiovascular, metabolic, and hepatic dysfunction. Unlike traditional biomarkers that reflect isolated pathways, ADMA reflects impaired endothelial homeostasis, which is central to both atherosclerosis and metabolic disease [21]. The association between elevated ADMA levels and clustering of cardiometabolic risk factors supports its role as a marker of cumulative risk [22]. Furthermore, its relationship with MASLD suggests that ED may contribute to hepatic steatosis through mechanisms involving insulin resistance, oxidative stress, and altered microcirculation. From a clinical perspective, the identification of candidate threshold values for ADMA (e.g., >160 ng/mL) is of exploratory interest. Such thresholds may, pending prospective validation, eventually facilitate risk stratification and earlier identification of patients at high risk for combined cardiometabolic and ED. When integrated with simple metabolic indices such as the TyG index and TG/HDL-C ratio, ADMA may in future enhance predictive accuracy, but at this stage these thresholds should be regarded as hypothesis-generating rather than ready for use in personalized management decisions.

### 4.3. Implications for Primary Care

Given that CAD patients are frequently managed in primary care settings, the ability to identify high-risk individuals using accessible biomarkers is of considerable importance [23]. The high prevalence of MetS and MASLD observed in this study suggests that routine assessment of metabolic and hepatic status should be incorporated into standard care for patients with CAD [24]. This is consistent with established cardiometabolic risk-assessment and prevention frameworks developed for related high-risk populations, such as patients with type 2 diabetes mellitus [25]. In this context, ADMA has potential utility as part of a multimarker approach to risk stratification. Its measurement may help identify patients with subclinical ED who would benefit from more intensive lifestyle modification, metabolic control, and pharmacological intervention. Importantly, such an approach aligns with the preventive focus of family medicine, emphasizing early detection and integrated care.

### 4.4. Comparison with Previous Studies

Previous studies have established the role of ADMA as a marker of ED and cardiovascular risk [26,27]. However, most investigations have focused on isolated associations with either vascular or metabolic parameters [28]. This is consistent with the broader rationale for developing biomarkers capable of detecting subclinical vascular damage before overt cardiovascular events occur [29]. The present study extends these findings by demonstrating the relationship of ADMA with a broader spectrum of cardiometabolic and hepatogenic disturbances within a unified clinical framework. Additionally, while the association between MASLD and cardiovascular disease has been increasingly recognized, the underlying mechanisms remain incompletely understood. Our findings support the hypothesis that ED, as reflected by ADMA, may represent a key mechanistic link between hepatic and cardiovascular pathology.

It should be noted, however, that the literature on ADMA as a cardiovascular biomarker is not uniformly consistent. A large meta-analysis of prospective studies found that, while ADMA was independently associated with all-cause mortality and incident cardiovascular disease across most cohorts, the strength and significance of these associations varied considerably by population and methodology; notably, some cohorts, including the Framingham Offspring Study, found ADMA predictive of mortality but not of incident cardiovascular events [30]. Similarly, in a large prospective cohort of patients with peripheral arterial disease, elevated ADMA was significantly associated with cardiovascular events and all-cause mortality but showed no significant association with cerebrovascular events [31]. At the metabolic level, at least one study in obese premenopausal women found no significant association between ADMA and cardiovascular risk factors, including blood pressure, insulin sensitivity, and lipid profile [32], in contrast to the robust associations with obesity and metabolic dysfunction observed in the present cohort. These discrepancies likely reflect differences in study population (general population versus disease-specific cohorts), outcome definitions (mortality versus incident events), and ADMA assay methodology, and they indicate that the associations reported here, while consistent with the broader weight of evidence, should not be assumed to generalize uniformly across all populations or outcome types.

This is consistent with recent narrative reviews of metabolomic and proteomic biomarkers in cardiology, which have highlighted methylarginine derivatives, alongside classical markers such as troponin and natriuretic peptides, as part of a broader panel of novel biomarkers reflecting inflammation, vascular remodeling, and metabolic disturbance in cardiovascular disease [33]. Reviews emphasize that cardiovascular pathogenesis is underpinned by common systemic processes, including ED and metabolic disturbance, which collectively drive structural and functional myocardial decline [34], reinforcing the rationale for evaluating ADMA as an integrative marker spanning vascular and metabolic domains rather than a single-pathway biomarker.

Endothelial dysfunction is not specific to the coronary or hepatic circulations: comparable mechanisms of impaired nitric-oxide-mediated vasodilation and vascular remodeling have been described in the pulmonary circulation [35], reinforcing the concept of endothelial dysfunction as a systemic process. Similarly, inflammation is now recognized as a shared driver linking hypertension, subclinical atherosclerosis, and cardiovascular risk more broadly [36], consistent with the multi-pathway framework proposed here for ADMA. Because most patients in this cohort had at least one cardiometabolic comorbidity, these findings should also be considered alongside contemporary cardiovascular prevention guidance for patients with concomitant type 2 diabetes, which likewise emphasizes an integrated, multi-marker approach to residual risk [37].

### 4.5. Strengths and Limitations

The strengths of this study include its cross-sectional, comprehensive assessment of cardiometabolic and hepatic parameters, and the use of multivariate analysis to identify independent predictors. The inclusion of patients with early-stage CAD (functional class I–II) enhances the clinical relevance of the findings for preventive strategies. However, several limitations should be acknowledged. First, and most importantly, the cross-sectional design means that the associations reported here—including the correlations between ADMA and metabolic indices, the ADMA—ED odds ratios, and the ROC-derived cut-offs—reflect concurrent associations rather than diagnostic or prognostic utility; the direction of causality between ADMA elevation, ED, and MASLD cannot be established, and these findings should be regarded as hypothesis-generating pending confirmation in prospective longitudinal cohorts. Second, some of the odds ratios reported (for example, OR 114.8 for overt ED at ADMA >160 ng/mL) had very wide confidence intervals, reflecting small cell counts in some strata; these estimates should be interpreted as indicating the direction and strength of association rather than a precise, reproducible effect size, and will require replication in larger samples. Third, although baseline pharmacological treatment (statins, antiplatelet agents, antihypertensive and glucose-lowering therapies) is now reported descriptively (Table 2), these medication classes were not entered as covariates in the regression models presented, and these agents are known to influence endothelial function and metabolic parameters; residual confounding by treatment cannot be excluded. Fourth, the study was conducted at two centers within a single city and geographical region (Tashkent, Uzbekistan), and the study population was relatively homogeneous with respect to age range (35–70 years), ethnicity, and healthcare setting; this homogeneity, together with the single-region recruitment, may limit the generalizability of the findings to other populations, healthcare settings, and more demographically diverse cohorts. Fifth, although ADMA was evaluated as a key biomarker, longitudinal clinical outcomes (e.g., cardiovascular events, progression of hepatic fibrosis) were not assessed. Sixth, this study lacked external validation: the candidate ADMA thresholds (e.g., >160 ng/mL for overt ED; 396.3 ng/mL for metabolic dysfunction) and the ROC-derived cut-offs for the TyG index and TG/HDL-C ratio were derived and evaluated within this single derivation cohort, and their diagnostic performance was not tested in an independent validation dataset; as such, these thresholds should be regarded as preliminary until confirmed in external cohorts. Seventh, the logistic regression models—including the estimate of OR 114.8 for overt ED at ADMA >160 ng/mL—included only cardiometabolic and anthropometric candidate predictors and were not adjusted for age, sex, smoking status, or renal function, all of which are recognized determinants of both endothelial function and ADMA metabolism; residual confounding by these unmeasured covariates cannot be excluded, and the reported associations should be interpreted as unadjusted for these factors. Eighth, the control group of 40 apparently healthy individuals was recruited to characterize a reference population but was not incorporated into the primary comparative analyses reported here; comparative evaluation of ADMA, FMD, and metabolic parameters between the CAD cohort and this healthy reference group was beyond the scope of the present cross-sectional analysis and is planned for separate reporting. Finally, we did not formally test whether ADMA adds incremental discriminatory value beyond established, low-cost metabolic indices (TyG index, TG/HDL-C ratio, BMI); such comparisons (e.g., nested regression models, DeLong’s test for AUC differences, or net reclassification improvement) require adequately powered, purpose-designed analyses and should be a priority for future prospective studies before ADMA can be recommended as an adjunct, rather than a substitute, for these simpler markers. Future prospective, multicenter studies with longitudinal follow-up, adjustment for baseline treatment, and independent external validation are needed to determine whether ADMA has genuine diagnostic value, and to validate the candidate ADMA thresholds proposed here, before any recommendation for use in risk stratification or clinical decision-making.

## 5. Conclusions

This cross-sectional study demonstrates that ADMA is significantly elevated in patients with stable CAD and is closely associated with a high burden of cardiometabolic risk factors, MetS, and MASLD. These findings support the role of ADMA as an integrative biomarker that captures the convergence of endothelial, metabolic, and hepatic dysfunction within a unified pathophysiological framework.

Among the studied cohort, MetS was present in 62.4% of patients and MASLD in 48.3%, underscoring the high prevalence of systemic metabolic dysregulation in this population. Elevated ADMA concentrations were independently associated with obesity, dyslipidemia, impaired glucose metabolism, and the clustering of cardiometabolic risk factors. Multivariable analysis identified obesity, type 2 diabetes mellitus, dyslipidemia, and the accumulation of three or more cardiometabolic risk factors as independent predictors of MASLD, highlighting the critical role of risk factor aggregation in hepatic involvement.

A significant dose–response relationship was observed between circulating ADMA concentrations and ED severity, as assessed by flow-mediated dilation. ADMA concentrations exceeding 160 ng/mL were associated with a markedly elevated odds of overt ED (OR 114.8; 95% CI 6.8–1935.0); given the small number of patients in the reference category, this estimate is imprecise and should be regarded as indicative of direction and strength of association rather than a stable effect size, pending confirmation in larger cohorts, while the diagnostic threshold of 396.3 ng/mL demonstrated meaningful discriminatory capacity for identifying metabolic dysfunction (sensitivity 52.7%, specificity 76.8%). Furthermore, metabolic indices including the TyG index and the TG/HDL-C ratio exhibited significant correlations with ADMA levels, with the TyG index demonstrating the strongest predictive performance (AUC 0.76; 95% CI 0.69–0.83).

Collectively, these cross-sectional findings position ADMA as a biologically plausible, hypothesis-generating integrative biomarker with potential future utility in risk stratification for patients with chronic coronary syndromes, rather than as an already validated diagnostic or prognostic tool. Its measurement, particularly in conjunction with readily available metabolic indices, may eventually help identify individuals with subclinical ED and high cardiometabolic burden, but this application requires prospective confirmation. Future longitudinal and interventional studies are warranted to establish the prognostic value of ADMA-based risk stratification and to assess the impact of therapeutic modulation of ADMA levels on cardiovascular and hepatic outcomes in this high-risk population before any clinical application is advocated.

## Figures and Tables

**Table 1:** Baseline demographic and clinical characteristics of patients with CAD according to MASLD status.

Variable	Overall (*n* = 298), %	Main Group (CAD + MASLD), *n* = 144 (%)	Comparison Group (CAD), *n* = 154 (%)	*p*-Value
Age, years	58.04 ± 0.81	58.14 ± 0.82	57.95 ± 0.80	0.08
Male sex, *n* (%)	122 (40.9%)	48 (33.3%)	74 (48.1%)	0.01
Female sex, *n* (%)	176 (59.1%)	96 (66.7%)	80 (51.9%)	
Education level				
Secondary education, *n* (%)	150 (50.3%)	68 (47.2%)	82 (53.2%)	0.47
Higher education, *n* (%)	148 (49.7%)	76 (52.8%)	72 (46.8%)	
Place of residence				
Urban, *n* (%)	176 (59.1%)	90 (62.5%)	86 (55.8%)	0.40
Rural, *n* (%)	122 (40.9%)	54 (37.5%)	68 (44.2%)	
Employment status				
Employed, *n* (%)	190 (63.8%)	88 (61.1%)	102 (66.2%)	0.63
Retired, *n* (%)	98 (32.9%)	50 (34.7%)	48 (31.2%)	
Disabled, *n* (%)	10 (3.4%)	6 (4.2%)	4 (2.6%)	
Current smoking, *n* (%)	16 (5.4%)	2 (1.4%)	14 (9.1%)	0.004
Alcohol consumption, *n* (%)	38 (12.8%)	0 (0%)	38 (24.7%)	0.001
Hypertension status				
Controlled normotension, *n* (%)	154 (51.7%)	70 (48.6%)	84 (54.6%)	0.61
Grade I hypertension, *n* (%)	76 (25.5%)	38 (26.4%)	38 (24.7%)	0.79
Grade II hypertension, *n* (%)	68 (22.8%)	36 (25.0%)	32 (20.7%)	0.41
Grade III hypertension, *n* (%)	0 (0%)	0	0	–
Systolic blood pressure, mmHg	122.53 ± 1.54	126.88 ± 1.38	118.18 ± 1.70	0.001
Diastolic blood pressure, mmHg	76.15 ± 0.97	76.53 ± 0.90	75.78 ± 1.03	
Left ventricular ejection fraction, %	62.45 ± 0.63	63.06 ± 0.35	61.84 ± 0.91	

“–” indicates that no statistical comparison was possible because no cases were observed in any group.

**Table 2:** Baseline pharmacological treatment in patients with CAD (*n* = 298).

Pharmacological Group	Patients, (*n* = 298)	%	*p*-Value
Antiplatelet therapy	242	81.21	<0.001
Statins	194	65.1	<0.001
ACE inhibitors	114	38.26	<0.001
Beta-blockers	198	66.44	<0.001
Calcium channel blockers	98	32.89	<0.001
Renin–angiotensin system blockers	76	25.5	<0.001
Diuretics	108	36.24	0.906
Thiazide/thiazide-like diuretics	62	20.81	<0.001
Long-acting nitrates	124	41.61	0.004
Glucose-lowering agents	84	28.19	<0.001
SGLT2 inhibitors	22	7.38	<0.001
Metabolic cardioprotectors	104	34.9	<0.001
Hepatoprotectors	84	28.19	<0.001

**Table 3:** Clinical characteristics of patients with CAD according to MASLD status.

Variable	Overall (*n* = 298)	Main Group (CAD + MASLD), *n* = 144	Comparison Group (CAD), *n* = 154	*p*-Value
CAD Functional Class I, *n* (%)	8 (2.7%)	8 (5.6%)	0 (0%)	0.003
CAD Functional Class II, *n* (%)	290 (97.3%)	136 (94.4%)	154 (100%)	0.05
Duration of CAD, years	4.07 ± 0.14	4.03 ± 0.15	4.10 ± 0.12	
Heart rate, beats/min	70.2 ± 8.2	70.4 ± 8.6	70.1 ± 7.9	0.08
Angina pain intensity (VAS score)	4.4 ± 1.2	4.8 ± 1.2	4.1 ± 1.1	0.002
Angina episodes per week	1.8 ± 1.3	2.2 ± 1.4	1.5 ± 1.2	0.004
Mean duration of angina episodes, min	6.2 ± 2.2	6.8 ± 2.3	5.6 ± 2.0	0.006
Mean angina-free walking distance, m	346 ± 91	310 ± 85	380 ± 95	0.001
Exercise tolerance on bicycle ergometry, W	83 ± 14	68 ± 12	98 ± 15	0.001
Ischemic ST-segment depression, *n* (%)	174 (58.4%)	96 (66.7%)	78 (50.6%)	0.006
Requirement for short-acting nitrates, doses/week	1.8 ± 1.1	2.6 ± 1.2	1.0 ± 0.7	0.004
NYHA Functional Class I, *n* (%)	128 (43.0%)	52 (36.1%)	76 (49.4%)	0.11
NYHA Functional Class II, *n* (%)	170 (57.0%)	92 (63.9%)	78 (50.6%)	

**Table 4:** Prevalence of MetS components in patients with CAD (*n* = 298).

MetS Component	Patients, *n*	%	χ^2^	*p*-Value
Abdominal obesity	214	71.81	28.356	<0.001
Triglycerides > 1.7 mmol/L	100	33.56	16.114	<0.001
Low HDL-C level	184	61.74	8.221	0.004
Arterial hypertension	174	58.39	4.195	0.041
Fasting glucose ≥ 5.6 mmol/L or type 2 diabetes mellitus	114	38.26	8.221	0.004
MetS (≥3 criteria)	186	62.42	9.188	0.002

**Table 5:** Anthropometric characteristics of patients in the main group (CAD + MASLD) and comparison group (CAD).

Parameter	Main Group (CAD + MASLD), *n* = 144	Comparison Group (CAD), *n* = 154	*p*
Body weight, kg	90.6 ± 2.18	79.97 ± 1.0	<0.05
Height, cm	164.46 ± 0.86	166.09 ± 0.7	<0.001
BMI, kg/m^2^	33.6 ± 0.85	28.99 ± 0.32	<0.05
Degree of obesity, *n* (%)			
Overweight (25–29.9)	48 (33.33%)	88 (57.14%)	χ^2^ = 38.364; *p* = 0.001
Grade I (30–34.9)	48 (33.33%)	64 (41.56%)	
Grade II (35–39.9)	30 (20.83%)	2 (1.30%)	
Grade III (≥40)	18 (12.5%)	0 (0%)	
Waist circumference, cm	89.58 ± 0.51	88.45 ± 0.6	0.054
Hip circumference, cm	97.41 ± 0.55	96.65 ± 0.62	–
WHR (waist-to-hip ratio)	0.92 ± 0.00	0.92 ± 0.00	–
Abdominal obesity, *n* (%)	114 (79.17%)	100 (64.94%)	0.054

“–” indicates that no statistical comparison was performed because no cases were observed in any group.

**Table 6:** Comparative hepatic ultrasonographic findings according to MASLD status (*n* = 298).

Parameter	Main Group (CAD + MASLD), *n* = 144	Comparison Group (CAD), *n* = 154	*p*
Right lobe craniovertical dimension, mm	140.33 ± 3.93	137.36 ± 2.06	<0.05
Left lobe craniocaudal dimension, mm	90.17 ± 0.81	83.14 ± 0.47	<0.001
Right lobe thickness, mm	111.36 ± 0.58	101.35 ± 0.31	<0.001
Left lobe thickness, mm	63.33 ± 0.42	56.0 ± 0.23	<0.001
Portal vein diameter, mm	10.68 ± 0.06	9.65 ± 0.02	<0.001
Inferior vena cava diameter, mm	16.12 ± 0.17	15.20 ± 0.06	<0.05
Hepatic artery diameter, mm	3.69 ± 0.01	3.5 ± 0.00	<0.05

**Table 7:** Association Between Serum ADMA concentrations and endothelial dysfunction severity in patients with CAD.

ADMA Category	ADMA Concentration (ng/mL)	Endothelial Function (FMD%)	OR	95% CI	*p*-Value
Normal (Reference Category)	≤120	≥10% (Normal endothelial function)	1.0	Reference	–
Moderately Elevated (Subclinical ED)	121–160	7.0–9.9%	8.6	1.8–41.2	<0.01
Highly Elevated (Overt ED)	>160	<6.9%	114.8	6.8–1935.0	<0.001

**Table 8:** ADMA level distribution in patients with CAD, according to the presence of MASLD.

Parameter	Flow Mediated Dilation (%)	Main Group (CAD + MASLD), *n* = 144		Comparison Group (CAD), *n* = 154	
		abs	M%	abs	M%
Normal (≤120 ng/mL)	≥10%	0	0	12	7.79%
Moderately elevated (121–160 ng/mL)	7.0–9.9%	10	6.94%	48	31.17%
Highly elevated (>160 ng/mL)	<6.9%	134	93.06%	94	61.04%
Within-group distribution (χ^2^)		χ^2^ = 53.389; *p* < 0.001		χ^2^ = 32.909; *p* < 0.001	
Between-group comparison (Pearson χ^2^)		χ^2^ = 21.814; *p* < 0.001			

## Data Availability

The data presented in this study are available on request from the corresponding authors due to privacy or ethical restrictions.

## Abbreviations

The following abbreviations are used in this manuscript:

AASLD	American Association for the Study of Liver Diseases
ACE	Angiotensin-converting enzyme
ADMA	Asymmetric dimethylarginine
AGAT2	Alanine-glyoxylate aminotransferase 2
AUC	Area under the curve
BMI	Body mass index
CAD	Coronary artery disease
CI	Confidence interval
DDAH	Dimethylarginine dimethylaminohydrolase
DMGV	Dimethylguanidino valeric acid
ED	Endothelial dysfunction
ELISA	Enzyme-linked immunosorbent assay
FIB4	Fibrosis-4 index
FMD	Flow-mediated dilation
HbA1c	Glycated hemoglobin
HDL-C	High-density lipoprotein cholesterol
L-NMMA	NG-monomethyl-L-arginine
MASLD	Metabolic Dysfunction-Associated Steatotic Liver Disease
MetS	Metabolic syndrome
NO	Nitric oxide
NOS	Nitric oxide synthase
NYHA	New York Heart Association
OR	Odds ratio
PRMTs	Protein arginine methyltransferases
ROC	Receiver operating characteristic
SDMA	Symmetric dimethylarginine
SGLT2	Sodium-glucose cotransporter-2
TG/HDL-C	Triglyceride-to-HDL cholesterol ratio
TyG	triglyceride–glucose index
